# Do peer-based education interventions effectively improve vaccination acceptance? a systematic review

**DOI:** 10.1186/s12889-023-16294-3

**Published:** 2023-07-14

**Authors:** Elisa L. S. Gobbo, Claudia Hanson, Khadija S. S. Abunnaja, Sibylle Herzig van Wees

**Affiliations:** Department of Global Public Health, Karolinska Institutet, Solna, 171 65 Sweden

**Keywords:** Vaccine hesitancy, Vaccine acceptance, Peer, Education, Intervention, Systematic review

## Abstract

**Background:**

Vaccination efforts are a vital part of controlling the spread of diseases, however, lack of vaccine acceptance undermines the efficacy of this public health effort. Current evidence suggests that the most effective interventions to support vaccination uptake and positive vaccination beliefs are multicomponent, and dialogue based. Peer-based education interventions are such a strategy that involves an individual within the same group to act as the vaccine educator.

**Objective:**

This review aims to consolidate the quantitative evidence surrounding the effectiveness and experience of peer-based education initiatives to improve vaccination beliefs and behaviors.

**Methods:**

We conducted a systematic search of PubMed, Web of Science, and a hand reference search. The search was conducted between April and June 2022. The inclusion criteria encompassed using peers, being education based, and being an intervention that addresses vaccination beliefs and behaviors (e.g. vaccination uptake).

**Results:**

Systematic screening revealed 16 articles in the final review. Half of the studies focused on students as their study population. The human papillomavirus vaccine was the most common vaccine assessed in the studies, followed by COVID and influenza vaccines. 11 out of 16 of the articles reported a positive impact of the peer intervention and two studies had mixed results. Six studies suggest a mixed peer- healthcare expert approach.

**Conclusions:**

Despite reported positive effects of using peer-education based initiatives to improve vaccine uptake and beliefs, this systematic review reveals that there is limited existing research in support of this strategy. The strategies that initially appear the most effect are those with a combined peer and health-expert approach, and those that have more group specific and long-term peer interventions. More research is needed to confirm these results and to assess the effectiveness of a peer-based education intervention in a wider variety of settings and for other vaccine types.

**Supplementary Information:**

The online version contains supplementary material available at 10.1186/s12889-023-16294-3.

## Introduction

Vaccines are indispensable for reducing disease morbidity and mortality. Low vaccine uptake and limited confidence in vaccines harms this endeavor [1]. The COVID-19 pandemic illustrated this problem. Based on surveys of general public opinions, rates of COVID-19 vaccine acceptance vary from 97.0% in Ecuador to as low as 23.6% in Kuwait [2]. While the rates of COVID-19 vaccine uptake have been making headlines, limited confidence in vaccines is a well-established phenomenon that applies to almost all vaccines [1]. This topic has been widely discussed within the vaccine hesitancy literature.

Vaccine hesitancy is a complex phenomenon that MacDonald (2015) described as the delay in acceptance or refusal of vaccines despite availability of vaccination services [3]. This definition has been challenged by H Larson (2022), M Goldberg (2021), and Bussink-Voorend et al. (2022) who propose that vaccine hesitancy should be defined as “a state of indecisiveness regarding a vaccination decision” [4–6]. Whilst MacDonald defines vaccine hesitancy as a behavior, the latter highlight it as a process of decision making [3]. Much research focuses on vaccine confidence or vaccine acceptance as more tangible and positive approaches [7]. Scholars have formulated a vaccine hesitancy determinants matrix that includes contextual influences, individual and group influences, and vaccine specific issues [3]. Overcoming these complex, historical, political and socio-cultural factors is not a simple task, and addressing them requires various interventions [8]. This systematic review includes studies that focus on vaccine hesitancy, vaccine acceptance, vaccine knowledge and beliefs, vaccine confidence and vaccination uptake. We recognize the diversity within these definitions and concepts, and the limits of them to fully capture vaccine knowledge, beliefs, and behavior. For the purpose of this review, we refer to vaccine beliefs and uptake to capture a broad range of widely discussed definitions.

Peer-based education interventions are refined and population specific interventions with the potential to increase vaccine uptake [9–11]. This strategy allows for improved cultural competencies taking into account many sociocultural and population characteristic factors [10, 11]. Prior studies have shown the benefits of peer-based interventions for improving other health behaviors [12, 13]. However, despite the mention of peer-based education in several reviews of vaccine beliefs and uptake[9, 10] to our knowledge, there is no comprehensive review of the effectiveness of and experience with peer-based education interventions for vaccination. Jama et al. postulate that peer-to-peer interventions could be a strategy for overcoming some of the barriers to vaccine uptake as the peers can lead by example and act as vaccine ambassadors [14]. There is also some evidence to show that peers in community-based intervention can improve vaccine coverage [15, 16]. This review aims to summarize the quantitative evidence surrounding the effectiveness and experience of peer-education initiatives that have been implemented.

## Methods

### Search strategy

For the systematic review, we conducted searches of Web of Science and PubMed. The review search was conducted between April and June 2022, then again in June 2023. We ran the searches with all of the combined search terms using the Boolean Operators. For Web of Science, all terms were searched with the ALL category, and for PubMed the advanced search setting was used with All fields. The search terms used for both databases were (Vaccine), (Vaccin*), (Vaccine hesitancy), (vaccine confidence), (vaccine coverage), (vaccination refusal), (vaccine-preventable diseases), (immunization), (peer education initiatives), (peer group*), (peer education), (Peer-to-peer), (peer-to-peer support), (peer), (health education), (vaccin* education), (education*), (Health knowledge, attitudes, practices), (patient education), (intervention). In addition, to the database searching, a hand search of article references was conducted. In relevant systematic reviews and pertinent articles, a researcher did an initial screening of the references to find other articles for inclusion. From the results of the database search, we first did a duplicate deletion using the EndNote software [17]. Then two members of the research team determined inclusion for the screening process. Two members of the team screened the articles that were sought for retrieval.

In the literature there is no single definition of a peer-based education intervention. For the purpose of this study, we define a peer as individuals with key shared characteristics, circumstance or experiences, and who do not have professional training [18]. Simoni et al. (2011) make a distinction between a peer intervener who acts to improve health behavior and has shared characteristics, rather than the colloquial peer as someone with equal standing [18]. For the purpose of this paper, “peer” will mean a “peer intervener.” In the case of a peer-based health intervention, they frequently collaborate with more qualified service providers [18]. Peer’s roles might include advocating, connecting people to resources, conveying information, and offering assistance [18]. Further, the majority of peer-based interventions include some kind of educational delivery. The core idea is that peer education is responsive to the values and objectives of the target group [18]. Peer-based education interventions allow for an individual affected by the same disease or among the same social group to provide vaccine and health information to peers in a more culturally or socially relevant manner than a health educator or provider [19, 20].

The guiding criteria for inclusion was that the articles need to have a peer-based education intervention on vaccination uptake and beliefs. Articles included had the term peer described above [18]. We defined an education intervention as an effort to improve knowledge or awareness on vaccination by providing some type of education via lecture, paper or digital information, tabling event, group meetings, or other means [18, 21]. We included articles that assessed an intervention that focused on addressing vaccination uptake and beliefs as their outcomes. This includes articles that addressed vaccine uptake, willingness-to-vaccine, vaccine hesitancy, and measuring knowledge and beliefs of vaccines.

Each screener assigned the article with a level; definitely fits, probably fits, most likely does not fit, and most likely excluded. This was a self-developed screening tool to guide the screening process. The articles screened as definitely fits and most likely excluded by both screeners were then directly included or excluded. Then based on discussion and input from a third member of the team, articles in probably fits or most likely does not fit were determined for inclusion [17, 21]. Having two options in the middle allowed for the researchers to have more nuance in identifying the article’s likelihood of inclusion and assisted with the conversations with the third team member.

In June 2023, the search was rerun on both PubMed and Web of Science to check for articles published in the year since the initial search. The same search terms and inclusion/exclusion criteria were utilized. On PubMed, the filter of article published last year was used to assess for new articles, and for Web of Science the filter for articles published in 2022 or 2023 was used.

### Data extraction and data analysis

For the data extraction, we modified a standardized systematic review extraction form [22]. One researcher conducted the extraction, and a second researcher double-checked the extraction results. Any discrepancies in data extraction were discussed among the authors and adjustments were made based on agreed upon reading or interpretation of the articles.

### Quality assessment

We utilized the Effective Public Health Practice Project quality assessment tool for quantitative studies to assess all included articles for their quality [23]. Articles were scored as 1 – Strong, 2 – Moderate, 3 – Weak based on assessment of study design, methods used, biases, and more (Additional file 1)[23]. Two researchers conducted the quality assessment and then matched the two scores. Our average quality score was 1.63 (Table 1) [22]. This indicates that there was a a moderate quality of research papers presented in this review. Overall, the many aspects of the study designs and testing were of high quality, but due to the nature of the interventions often participant randomization or blinding were not conducted [23].

**Table 1:** Summary of articles

Title	Authors	Year	Location	Study Aim	Study Population	Vaccination Type	Intervention	Methods Used	Primary Outcome Measures	Results Recap	Quality Score
Development and acceptability of a peer-paired, cross-cultural and cross-generational storytelling HPV intervention for Korean American College Students	Minjun Kim, Haeok Lee, Peter Kiang, Jeroan Allison	2019	US	Reports on the development of a cross-cultural, cross-generational story-telling HPV intervention using a peer-paired method.	Korean American college women	HPV	Peer-paired cross-cultural and cross-generational story telling	RCT	Self-satisfaction and endorsement of the storytelling HPV videos.	Intervention group had higher satisfaction. Suggests that it would be important to include both peer stories and health provider messaging.	1
A Peer-Based Educational Intervention Effects on SARS-CoV2 Knowledge and Attitudes among Polish High-School Students	Maria Ganczak, Oskar Pasek, Lukasz Duda-Duma, Julia Komorzycka, Karol Novak, Marvin Korzen	2021	Poland	Evaluate the impact of a peer-based educational intervention on COVID on knowledge and attitudes on the pandemic.	Polish High School Students	COVID-19 vaccine	A peer based educational intiative.	Survey	Knowledge scores regarding COVID, attitude scores, and intention to vaccinate	Pre and post intervention the level of knowledge increased and improved attitudes	1
Increasing influenza and pneumococcal immunization rates: a randomized controlled study of a senior center-based intervention	J W Krieger, J S Castorina, M L Walls, M R Weaver, S Ciske	2000	US	A RCT of a senior-based peer-to-peer intervention to increase pneumococcal and influenza immunization rates.	Urban senior population (and pneumococcal)	Influenza	Intervention group received informational brochures (with reply cards on immunization status), calls from senior volunteers, and computerized immunization tracking.	Survey	% of those vaccinated with influenza and pneumococcal between the two years with the introduction of the intervention.	Compared with the control group, the intervention group had a greater increase in people with influenza and pnumococcal vaccination from the prior year.	2
A Randomized Controlled Trial of Group Well-Child Care: Improved Attendance and Vaccination Timeliness	Ada M Fenick, John M Leventhal, Walter Gilliam, Marjorie S Rosenthal	2020	US	To compare Group well-child care (GWCC) compared with individual well-child care.	Mother-infant dryads in New Haven	Childhood vaccines	Group well-child care (GWCC)	RCT	Visits attended, rates of admission to hospital or ED, vaccination timeline at 2,4, 6, and 12 months.	Infants in the GWCC were more likely to get immunization within 1 month of scheduled at 6 months and 12 months. However there was no significant effect for 2 and 4 month vaccinations.	1
Effect of Peer Education Knowledge of Human Papilloma Virus and Cervical Cancer among Female Adolescent Students in Benin City, Nigeria	Ayebo Sadoh, Chukwunwendu Okonkwobo, Damian Nwaneri, Bamidele Ogboghodo, Charles Erigiea, Osawaru Oviawe, OMolara FAmuyiwa	2018	Nigeria	Determine the effect of peer education on the knowledge of female adolescents about HPV, cervical cancer, treatment, and prevention.	Female adolescent students in Benin City	HPV	Trained students delivered messages on cervical cancer and HPV using fliers to their peers in a classroom setting.	Survey	Knowledge score on cervical cancer and prevention methods	Pre and post intervention the mean knowledge score increased. Compared with the school cohort (control), a much higher percentage of the students knew that PAP smears and vaccination could prevent cervical cancer seminar cohort	2
Effects of a narrative HPV vaccination intervention aimed at reaching college women: a random controlled trial	Suellen Hopfer	2011	US	To evaluate the effect of a narrative intervention aimed at increasing HPV vaccination among women	College women	HPV	Narrative messaging intervention with peer, medical expert, or combination intervention options	RCT	Likelihood to vaccine 2 months after the intervention.	The peer-expert narrative intervention had nearly double the vaccination of the control (22% to 12%)	2
Multi-level intervention to prevent influenza infections in older low income and minority adults	Jean Schensul, Kim Radda, Emil Coman, Elsie Vazquez	2009	US	To address persistent inequalities influenza vaccination in African American and Latino adults through a multi-level participatory intervention grounded in group and individual empowerment	African Americans and Latinos living in public senior housing	Influenza	A multi-level participatory intervention that was designed by the research team and participants from the housing units.	RCT	Observation of the intervention strategy and the increase in vaccination from the year before compared with the control building.	In the intervention buildings the vaccination rate increased from 30.4 to 71%. Whereas, in the control building there was only an 18% increase in vaccination.	2
Using Peer-to-Peer Education to Increase Awareness and Uptake of HPV Vaccine Among Chinese International Students	Aaron Esagoff, Samuel Cohen, Guoxuan Chang, Ozlem Equils, Sarah Van Orman, Alicia Burnett	2019	US	Examine the impact of peer to peer education program about HPV disease and vaccination among Chinese International Students	Chinese International Students at USC	HPV	Mandarin-speaking students volunteers as peer educations who were trained on HPV.	Survey	% reporting receiving th HPV vaccine, HPV knowledge, belief of likelihood to acquire HPV, interest in vaccination if it was free, and number of students that visited the health center for a vaccination.	On going project — of 400 students educated, 80 visited the health center. (Unknown vaccination status of the total 400)	2
Effect of peer-education on the willingness to vaccinate against COVID-19 among high school students	O. Pasek, J. Michalska, M. Piechowicz, M. Stolinski, M. Ganczak	2021	Poland	Assess the influence of peer-based education on willingness to vaccinate among Polish High school students	Polish High School Students	COVID-19 vaccine	Peer education campaign introduced into 24 school.	Survey	Willingness to vaccinate	Willingness to vaccinate grew from 31.8 to 35.2%.	1
Does bar-based, peer-led sexual health promotion have a community-level effect amongst gay men in Scotland?	Flowers, P; Hart, GJ; Williamson, LM; Frankis, JS ; Der, GJ	2002	Scotland	Evaluate the effectiveness of a bar-based, peer-led community intervention to promote sexual health among gay men.	Gay Men in Scotland	Hepatitis B virus (HBV)	Using the Gay Men’s Task Force (GMTF) which using peer-led and community level intervention	Survey	% who vaccinated post-introduction of the intervention compared with vaccinate rate at baseline	In regards to vaccination, the men who had contact with a peer educator in Glasgow reported 59% hep vaccination which is compared with 44% at baseline. In Edinburgh, vaccination increased from 53 to 56%. (Limitation, not all the same people)	2
Nudges for COVID-19 voluntary vaccination: How to explain peer information?	Shusaku Saski, Tomoya Saito, Fumio Ohtake	2021	Japan	This study aims to discover other-regarding information nudges that can reinforce intention to vaccinate, without impeding on autonomous decision making.	Japanese	COVID-19 vaccine	An online experiment with three treatment groups that different peer information; comparison, influence-gain, influence-loss and control. All three using the nudge theory.	RCT	Willing to receive free vaccine and willingness to pay level	With an influence gain messaging, the willingness to receive a vaccine increased to 91.5% and willingness to pay also increased.	3
Community health workers on a college campus: Effects on influenza vaccination	Huang, Jack J.; Francesconi, Maria; Cooper, Madeline H.; Covello, Allyson; Guo, Michelle; Gharib, Soheyla D.	2018	US	Assess the impact of a campus community health worker program on influenza vaccination.	Undergraduate students	Influenza	HealthPALs conducted in person outreach to different dormitories during flu clinics. Then also did personalized dormitory intervention the following year.	Population vaccination rate	Vaccination rate between the three different years.	Over the first year vaccination increased 66% and in the second year it grew by 85%	2
Pharmacy student involvement with increasing human papillomavirus (HPV) vaccination among international college students	Angela G.Long,Craig M.Roberts,Mary S.Hayney	2017	US	Determine if peer-to-peer HPV outreach and education held on UW campus could increase HPV vaccination by 20%.	International (specifically Chinese) undergraduate students	HPV	Peer-to-peer intervention using pharmacy students as the educators	Population vaccination rate	HPV vaccinations during the same time period the year prior.	With the intervention vaccination increased by 41% compared with the year before.	1
Engaging Same-Day Peer Ambassadors to Increase Coronavirus Disease 2019 Vaccination Among People Experiencing Unsheltered Homelessness in Los Angeles County: A Hybrid Feasibility-Evaluation Study	Chelsea L. Shover, Allison Rosen, José Mata, Brooke Robie, Julissa Alvarado, Ashley Frederes, Ruby Romero, Jacqueline Beltran, Anna Bratcher, Alicia H. Chang, Kristen R. Choi, Candelaria Garcia, Steven Shoptaw, Priyanka Guha, Lindsey Richard, Gunner Sixx, Angel Baez, Anthony Coleman, Sarah Harvell, Shirnae Jackson, Caroline Lee, Joanna Swan, Kenny Torres, Emily Uyeda Kantrim, Maya McKeever, Anh Nguyen, Adam Rice, Marisol Rosales, Jordan Spoliansky, Elizabeth Bromley, Heidi Behforouz, Lillian Gelberg, Pamina M. Gorbach, Anne W. Rimoin, and Emily H. Thomas	2022	US	Evaluate the feasibility and acceptabilty of engagign unhoused peer ambassadors for COVID-19 vaccination for the unhoused	People experiencing unshltered homelessnes	COVID-19 vaccine	PAs were using incombination with a vaccination event to share their experiences getting vaccinated	Mixed methods	Feasibility via number of Pas enrolled, acceptability via average number of hours a PA participated. Then did person-time calculation to calculate the number of additional clients vaccinated per hour of PA participation.	197 additional people were vaccinated at events using PAs over a total of 167 PA hours.	1
An online community peer support intervention to promote COVID-19 vaccine information among essential workers: a randomized trial	Dominic Arjuna Ugarte, Jeremy Linb, Tianchen Qian and Sean D. Younga	2022	US	Testing the efficacy of a 4-week online peer-led intervention to promote COVID-19 vaccine information requests	Essential workers	COVID-19 vaccine	Online peer leader based intervention with hestiant indivduals. Peer leaders ran a facebook group with peers to discuss vaccine information and doubts.	RCT	Number of people requesting vaccine information and odds of getting a vaccination	Intervention group had 6 people request information and 10 people get vaccinated, and in the control group 0 requested information and 6 got vaccinated.	1
Diné teachings and public health studetns informing peers and relatives about vaccine education: Providing Diné (Navajo)-centered COVID-19 education materials using student health messengers	Marissa Tutt, Chassity Begay, Shawndeena George, Christopher Dickerson, Carmella Kahn, Mark Bauer and Nicolette Teufel-Shone	2022	US	Adapt the health education material and conduct a retrospective pretest of the materials to assess participants attitudes and behavioral controls before and after the education session.	Diné	COVID-19 vaccine	Use trusted health messagers to address Diné adults vaccine concerns and hesitancy.	Survey	A finalized set of COVID-19 vaccine materials for the Diné community and the effictiveness of the intervention by assessing the attitudes, perceived behavioral controal, subjective nrms, and intent to receive the COVID-19 vaccine.	All participants indicated a change in the positve direction post educaiton. Statistically significant changes in participants who believed getting the COVID-19 vaccine was a good idea, that the vaccine coudl prevent COVID, and the vaccine would protect the community.	1

*This table includes the title, authors, year of publication, study location, study aim, study population, vaccination type(s) studied, the intervention type, the methodology strategy used, the primary outcome measure, a results recap, and the quality score determined by the authors

## Results

**Fig. 1:**
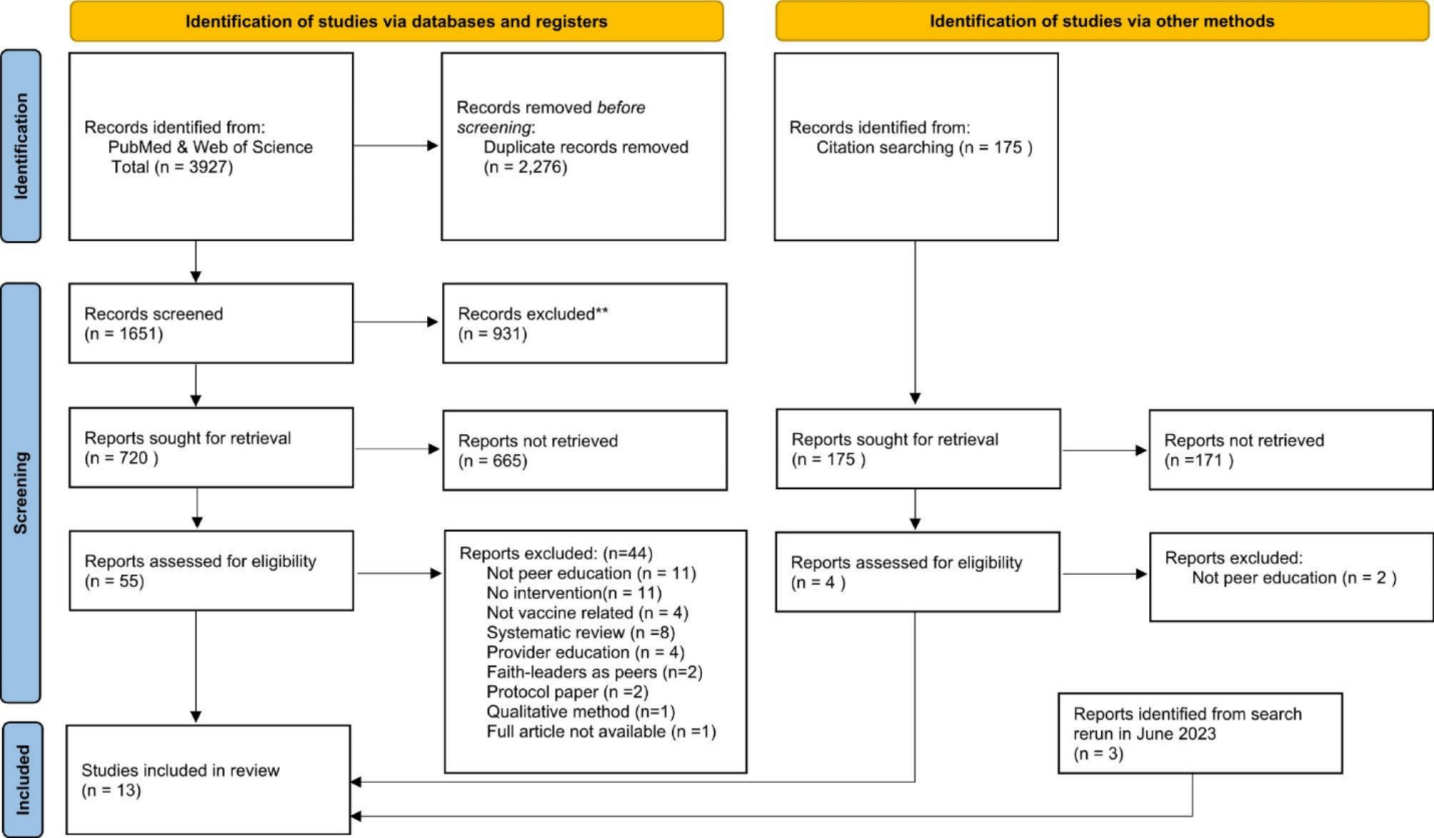
PRISMA flow diagram of systematic review. Strategy PRISMA 2020 flow diagram for new systematic reviews which included searches of databases, registers and other sources *From*: Page MJ, McKenzie JE, Bossuyt PM, Boutron I, Hoffmann TC, Mulrow CD, et al. The PRISMA 2020 statement: an updated guideline for reporting systematic reviews. BMJ 2021;372:n71. doi: 10.1136/bmj.n71. For more information, visit: http://www.prisma-statement.org/

The systematic review revealed 3927 articles from PubMed and Web of Science, and 175 from citation searching (Fig. 1). After removing duplicates, and screening for eligibility, 11 articles were included from database searching. In addition, two eligible articles from citation searching and three articles from the June 2023 search rerun were identified making a total of 16 eligible articles included in this review. One article was excluded due to inability to access the full article.

Table 1 summarizes all of the results. For the study populations, eight articles studied students, four of which focused on women, two studied senior citizens, and six studied other populations (parents, Japanese general population, gay men, essential workers, and the Diné/Navajo). Studies took place in five different countries with 11 in the United States, two in Poland, and one study in Japan, Scotland, and Nigeria. The vaccines assessed were COVID-19 (six), followed by HPV (human papilloma virus) (five), influenza (three), then childhood vaccination (one), and hepatis B virus (one). The various study methods utilized in the articles were surveys (seven), randomized control trials (six), population vaccination rate (two), and mixed methods with surveys and interviews (one).

The studies covered a variety of primary outcome measures as a strategy to assess a peer education-based intervention for vaccine beliefs and outcomes. Five of them measured a change in vaccination rate [24–28], four collected vaccination uptake rates [29–32], three gathered willingness to vaccinate [33–35], another three surveyed the knowledge and beliefs of participants [36–38], and one study assessed the satisfaction with the intervention [39]. Overall, 13 of 16 studies reported a positive impact of the peer intervention, in the studies’ outcome measure [24–28, 31, 33–39]. A positive impact could mean improved knowledge or intention to vaccine or improved vaccination rates or uptake of vaccines. Three studies had mixed results with some results showing improvement, but some outcomes not being statistically significant [30, 32, 40]. Six out of the 16 studies utilized or suggest a mixed peer and healthcare expert approach [25, 28, 31, 33, 36, 38–40].

The different intervention strategies used were grouped into a quick chat with a peer [6], a workshop/lecture approach [5], a narrative- onetime approach [3], and repeated contact with a peer [2]. The interventions utilizing a quick chat with a peer strategy involved peer education tables or promotions [26, 30], having peers provide information and support during an immunization clinic [27, 28, 31], and peer phone-calls to unvaccinated individuals [24]. Even though this was the least intensive intervention strategy, five studies had a positive impact on vaccination with increased vaccination rates compared with baselines [24, 26–28, 31]. Several other studies used a one-time workshop or lecture by peers to provide information on vaccination[34, 36–38, 40]. These mostly involved a peer training by public health professionals and then an educational session. All studies, except one [40], had positive impact on either knowledge and beliefs or willingness to vaccinate. The Fenick, et al. study had a mixed impact, but utilized a slightly different intervention strategy with group wellness visits with parents to encourage peer support for childhood vaccination [40]. Three studies used a narrative-onetime approach that had peer-based messaging provided online and assessed the impact on willingness to vaccinate, all of which had a positive impact [33, 35, 39]. Lastly, two studies used more intensives strategy with repeated peer contact. One was via a two-month long programing among seniors [25], and one had a peer led Facebook group among essential workers [32]. Both studies had an increase in vaccination uptake or interest in vaccination information.

Several studies (nine) showed improvements in the uptake of vaccines [24–28, 31, 33, 34, 38] [33]. In an intervention at a senior living center the immunization rates for influenza and pneumococcal rose by a greater percentage for those in the intervention building than the control over the two years [24]. Similarly, in a population of low income and minority adults with a repeated peer multilevel participatory intervention there was a 40.6% increase in the intervention group compared with only an 18% increase in the control group [25]. A narrative intervention to improve HPV knowledge and vaccination in college aged women, found that the combined peer and health expert intervention had a nearly double rate of HPV vaccination at two months compared with the control [39]. In a study that utilized peer ambassadors at same-day COVID-19 vaccination clinics, 197 more individuals were vaccinated at centers with peers compared with those without peers [31].

Peer intervention also appears to improve knowledge and intention to vaccinate. In a study of students regarding COVID-19, the intervention had a slight increased willingness to vaccinate from 31.8 to 35.2% [34]. After receiving messaging on HPV by trained students, female adolescence in Nigeria mean knowledge score increased from 12.94 to 53.74 [37]. Unlike the other studies, these two focused on vaccine knowledge and beliefs, rather than changes in vaccination uptake.

Some studies found a mixed result. In a study with a group-based general wellness checkup visit, compared with regular individual visits, they found that there was no difference between the two groups in terms of vaccination timeliness at 2 and 4 months. However, the infants were more likely to be immunized on schedule at 6 months and 12 months, and to attend all 6 visits [40]. A study conducted at a university among Chinese international students found limited knowledge levels on HPV, but 94.9% were interested in receiving the vaccine [28]. However, of the first 400 students educated only 80 actually visited the health center for vaccination [28]. Therefore, whilst the education was arguably successful, it did not lead to significant uptake of the HPV vaccine.

## Discussion

This systematic review of peer-education based interventions to improve vaccine uptake and beliefs revealed several benefits of peer-based interventions. Thirteen studies illustrated some positive effect of the peer-based education intervention on the intended outcomes [24–28, 33–37, 39]. This shows that the peer-education based interventions could be a useful tool for improving vaccine uptake and beliefs. Of the 13 studies reporting a positive effect, six combined the peer-based education intervention with a health expert intervention. The studies that were using mixed approaches demonstrated a stronger impact on their outcomes, than studies that only used one intervention type [25, 28, 33, 36, 39, 40]. Thus, future studies or interventions should consider using the potential benefits of utilizing a combination health expert and peers’ approach to encourage vaccination.

Furthermore, the different specific intervention strategies appear to affect the efficacy of the intervention. Although unable to directly compare results, there are some trends that can be seen in the review. Studies that only provided a quick chat with a peer intervener through tabling or a bar intervention seem to have the weakest impact on vaccination and vaccine knowledge [26–28, 30, 31, 35]. However, those at universities are more successful than others [27, 28]. The one-time workshop interventions seem to have some limited success in improving vaccine knowledge [34, 36]. The one-time narrative approaches seem be even a bit more effective in improving knowledge [37–39], and one even reported doubling the HPV vaccination rate among students receiving the intervention [33]. The two studies with repeated peer interventions had statistically significant increases in vaccination rates, indicating the strength of this strategy [25, 32]. This indicates that a more targeted and repeated approach, appear to have more potential than more generic, one-time interventions.

It is important to note that the peer education interventions presented in these studies were mostly used to encourage vaccination rather than to address vaccinate hesitancy or refusal. Seven of the studies focused on improving vaccine uptake or a change in vaccination rates among groups that were not yet vaccinated such as international students, children, or seniors [24–28, 32, 40]. Thus, the peer-based interventions show a generally positive effect on vaccine uptake and beliefs, but apart from one paper, - on the Diné (Navajo) population which is known to be vaccinate hesitant rooted in governmental mistrust [34] - there was no research on the effectiveness of peer-based educational interventions in communities that present low levels of vaccine confidence. This suggests that there is limited evidence for the use of peer- based interventions to address vaccine hesitancy, which is rooted in specific communities for example, migrant communities, anthroposophic or religious communities, or online communities.

### Limitations

There are limitations to the generalizability of these results to diverse range of population groups and vaccine types. The scope of the results is also restricted as only one study took place in a middle-income country [37], none in a low-income country, and the majority were set in the United States [24, 25, 27, 28, 30–33, 38–40]. Several United States-based studies (ten) demonstrated a positive impact, pointing to encouraging benefits of a peer intervention in a high-income setting amongst adults [24, 25, 27, 28, 31–33, 38–40]. However, the limited geographic scope curtails the generalizability of the evidence found in the systematic review. One study in Nigeria showed promising results with an illustrated improvement in awareness and knowledge regarding the HPV vaccine and cervical cancer. The study did not explore the impact on HPV uptake; thus, the effect remains poorly understood [37]. To understand the potential of peer-based education initiatives for different areas it is necessary to conduct more research in economically and geographically diverse regions.

Additionally, more long-term studies may be required to assess the sustainability of effects of peer-based education intervention. For example, two studies demonstrate the effects of a peer-education on either knowledge or vaccination rates for up to one year [24, 40]. For some vaccines that require a repeated yearly dosage, such as influenza, it may be pertinent to assess the effects of peer-based education interventions for more long-term sustainability.

There are several limitations to this systematic review. Publication bias could be a limitation of the results. We found no articles with a negative outcome. Research with less favorable results could have been conducted but not published. Some studies also could have been missed due to the utilization of different terminology other than peer. The quality of the studies found was mixed. One consistent issue throughout the studies was the inability to blind the participants due to the nature of the interventions. We also had to exclude one article due to an inability to access the full article.

### Recommendations

Based on the limitations of the results, we recommend further research to examine the full benefits of a peer-based education approach, particularly for different population groups (not only adults), vaccination types (particularly childhood vaccines), and geographical regions (particularly in low-income settings). Given that we found that the benefits of peer-based education intervention to address vaccine beliefs and uptake are inconclusive, we recommend further studies that apply strong methods such as randomized controlled trial. This would provide greater generalizability of the results and provide clearer guidance for policy making for public health promotion. A peer-based intervention may work better in certain setting than in others, but the positive nature of the results implies that the strategy should be proliferated more in research and interventions [8]. More studies into peer-based interventions with known vaccine hesitant populations would be useful to determine if they are useful for addressing specific vaccine fears or rumors and not just for encouraging vaccination. Also, conducting a randomized controlled trial comparing the effects of a single peer-based and a combined (health expert and peer) would be beneficial for strengthening the findings.

## Conclusion

In conclusion, there is limited existing research on peer-based education interventions to improve vaccine beliefs and uptake. The research that exists illustrates the promise of this approach for certain vaccines and populations. To fully assess the effectiveness of peer-based education further research into this strategy for different peer groups, in different parts of the world, and using different methodological approaches is required. Importantly, further research must examine peer-based education interventions in vaccine hesitant communities [8]. Implementing vaccine education by peers could help to address socio-cultural barriers through a culturally competent addition to traditional vaccine interventions. Whilst the claim of the value of peer-based education system continues to be made in the literature, more solid evidence on best approaches is needed.

## Electronic supplementary material

Below is the link to the electronic supplementary material.

Supplementary Material 1

## Data Availability

The datasets used and/or analysed during the current study is available from the corresponding author on reasonable request.

## Abbreviations

HPV: Human Papillomavirus
